# Tapping Into Actinobacterial Genomes for Natural Product Discovery

**DOI:** 10.3389/fmicb.2021.655620

**Published:** 2021-06-22

**Authors:** Tanim Arpit Singh, Ajit Kumar Passari, Anjana Jajoo, Sheetal Bhasin, Vijai Kumar Gupta, Abeer Hashem, Abdulaziz A. Alqarawi, Elsayed Fathi Abd_Allah

**Affiliations:** ^1^Department of Biosciences, Maharaja Ranjit Singh College of Professional Sciences, Indore, India; ^2^School of Life Sciences, Devi Ahilya Vishwavidyalaya, Indore, India; ^3^Departmento de Biología Molecular y Biotecnología, Instituto de Investigaciones Biomédicas, Universidad Nacional Autónoma de México, México City, Mexico; ^4^Biorefining and Advanced Materials Research Center and Center for Safe and Improved Food, Scotland’s Rural College (SRUC), SRUC Barony Campus, Dumfries, United Kingdom; ^5^Department of Botany and Microbiology, College of Science, King Saud University, Riyadh, Saudi Arabia; ^6^Department of Mycology and Plant Disease Survey, Plant Pathology Research Institute, Agricultural Research Center (ARC), Giza, Egypt; ^7^Department of Plant Production, College of Food and Agricultural Sciences, King Saud University, Riyadh, Saudi Arabia

**Keywords:** actinobacteria, genomics, antibiotics, secondary metabolites, biosynthetic gene cluster

## Abstract

The presence of secondary metabolite biosynthetic gene clusters (BGCs) makes actinobacteria well-known producers of diverse metabolites. These ubiquitous microbes are extensively exploited for their ability to synthesize diverse secondary metabolites. The extent of their ability to synthesize various molecules is yet to be evaluated. Current advancements in genome sequencing, metabolomics, and bioinformatics have provided a plethora of information about the mechanism of synthesis of these bioactive molecules. Accessing the biosynthetic gene cluster responsible for the production of metabolites has always been a challenging assignment. The genomic approach developments have opened a new gateway for examining and manipulating novel antibiotic gene clusters. These advancements have now developed a better understanding of actinobacterial physiology and their genetic regulation for the prolific production of natural products. These new approaches provide a unique opportunity to discover novel bioactive compounds that might replenish antibiotics’ exhausted stock and counter the microbes’ resistance crisis.

## Introduction

Actinobacteria are an omnipresent group of bacteria that play an essential part in recycling complicated organic matter in the soil. They are of great interest for researchers as they are versatile producers of diverse metabolites with several biotechnological applications. Genome mining tools could help the researchers to determine the BGCs of secondary metabolites compounds like herboxidiene, paulomycin, sceliphrolactam, bagremycin, and humidimycin produced from *Streptomyces* (Lee et al., 2020). Actinobacteria synthesize several secondary metabolites with various biological activities (Passari et al., 2017). Several phenolic compounds (catechin, kaempferol, chebulagic acid, chlorogenic acid, Asiatic acid, ferulic acid, arjunic acid, gallic acid, and boswellic acid) and paclitaxel, an anticancer compound were detected and quantified by *Streptomyces* species as reported by Passari et al. (2017). The majority of bioactive compounds from actinobacteria discovered to date have been derived from *Streptomyces* sp. It is estimated that *Streptomyces* genome can synthesize 100,000 antimicrobial metabolites from which only a speck has been identified (Doroghazi et al., 2014). For decades, actinobacteria have been intensively exploited by industrial discovery programs for novel natural products (NPs), to derive antibiotics currently in clinical use (Genilloud, 2018). Despite this, the development and spread of multi-drug resistance among pathogens have made it difficult to treat the infections resulting in significant health concerns. The lack of therapeutic solutions for the rising disorders by resistant pathogens in the clinic requires a new approach to responding to novel antibiotics. Tapping into the actinobacterial genomes and exploiting rare actinobacteria with unexpected genes might be an answer to the ever-rising need of novel antibiotics (Ward and Allenby, 2018). The rare actinobacteria which are of non-streptomycetes category, isolated from diverse habitats have efficiency for the discovery of novel bioactive compounds. The draft genome of *Saccharomonospora* sp. CNQ490 has revealed 19 unexplored BGCs exhibiting its diverse metabolic capacity. These actinobacteria can be a promising source for drug discovery (Luzhetskyy et al., 2007; Thumar et al., 2010; Hug et al., 2018). Particularly, non-streptomycetes belonging to genera, such as *Micromonospora*, *Nocardia, Actinomadura, Actinoplanes, Streptoverticilllium*, and *Saccharopolyspora* have been reported to produce unique antibiotics with various biological activities (Hug et al., 2018), such as abyssomicins (Bister et al., 2004) and proximicins (Fiedler et al., 2008) from *Verrucosispora* strains. Subsequently, approximately 2,250 new bioactive secondary metabolites have been discovered from rare actinobacteria according to 2005 information (Genilloud et al., 2011; Subramani and Aalbersberg, 2013).

**FIGURE 1:**
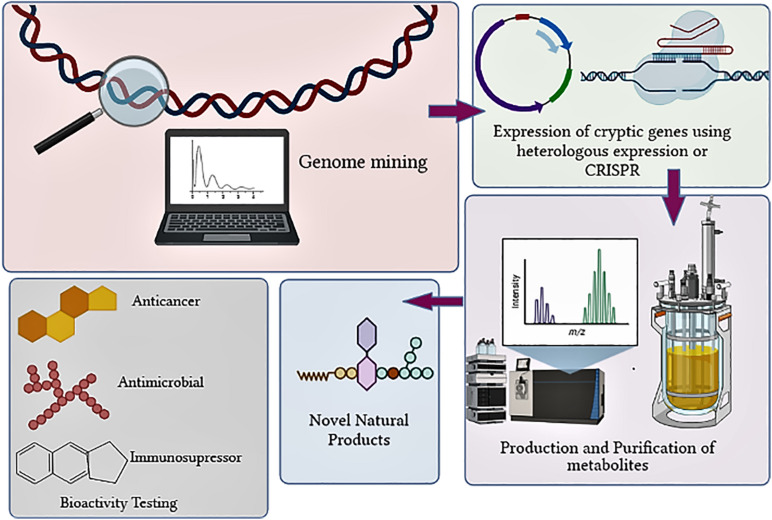
Overview of steps involved in discovery of novel NPs from actinobacteria.

In the past decade, the failure to unveil the full potential of the natural product (NP) producing actinobacteria was due to the lack of understanding of their genome and expression of biosynthetic gene clusters (BGCs). The advent of rapid and economical sequencing technologies has revealed that BGCs in actinobacteria are in more significant numbers than the already discovered molecules synthesized by them (Schorn et al., 2016; Tokovenko et al., 2016). The evolution of genomics and bioinformatics tools has exponentially increased the understanding of useful genetic information that can be exploited to discover bioactive compounds (Machado et al., 2015; Weber and Kim, 2016; Albarano et al., 2020; Figure 1). The genome sequencing of *Streptomyces* has revealed that they contain BGC in large numbers. *Streptomyces clavuligerus* was found to have 58 BGCs, *Streptomyces bottropensis* have 21 BGCs, whereas *S. avermitilis* contain 30 BGCs (Zhang et al., 2017). Actinobacterial genomes are sequenced rapidly since 2013 for the hunt for novel bioactive NP discovery. Till recently, a total of 1,749 *Streptomyces* genomes have been deposited as of the 6th of February 2020 and are available in the RefSeq database. The 1,749 *Streptomyces* genomes contains 867 contig level, 646 scaffold level, 36 chromosome level, whereas 200 complete genomes are included (Lee et al., 2020).

The current review explicates the recent advancements in actinobacterial genomic research. It gives an insight into the different technologies currently employed in genomics for NP discovery, along with challenges and research gaps that need to be filled for better outcomes.

## Bioinformatic Tools for Genome Mining in Actinobacteria

A plethora of actinobacteria genomes has been sequenced and submitted to databases by different researchers. RefSeq database contains 1,749 *Streptomyces* genomes (Lee et al., 2020). Researchers started mining genomes successfully and isolated potential metabolites from the available genetic data based on these available databases. Bioinformatics tools like antiSMASH, PRISM, ClustScan, and CLUSEAN have revolutionized the researchers’ genome mining experience and have acquainted us with the untapped potential of the actinobacteria. Over time, the actinobacterial strain collection from different habitats has increased, resulting in massive genetic data enhancement. This has resulted in an overwhelming discovery of novel metabolites in the past 10 years. Initially, genome mining was accomplished by detecting and exploiting the central enzymes of secondary metabolite production’s biosynthetic pathways. These enzymes include polyketide synthases (PKSs), non-ribosomal peptide synthetases (NRPs). Analyzing the massive database of actinobacterial genomics requires specialized bioinformatics tools.

Later, various computational tools like antiSMASH and PRISM were developed that could identify partially or complete BCGs. These tools efficiently compared the user-submitted genomes with the available BCGs database and provided a structural prediction of secondary metabolites. The methods used for structural prediction followed certain basic rules which involved consideration of substrate specificity of catalytic domain of PKS and NRPS module for developing the backbone of metabolite structure. It was followed by identification of the tailoring domains through which the further cyclization of compound was predicted and later mapped with the available databases to provide an idea of secondary structure produced by unknown BGCs. The prediction accuracy completely depends on the algorithm and available databases. Presently, the database comprises of limited number of experimentally validated genes. The incorrect gene annotations have hampered the accuracy in gene mining and prediction of structure of NPs and lead to spread of false functional role of the gene (Salzberg, 2019). Despite the advancements in structural predictions over time, lack of information on tailoring enzymes and assignment of related BGCs as hybrid BGCs still require validations and improvements. Certain bioactive compounds like streptoketides from *Streptomyces* sp. Tu6314, atratumycin from *S. atratus*, nybomycin from *S. albus* has been discovered using antiSMASH based genome mining (Sun et al., 2019; Qian et al., 2020). Bioinformatics tools like MultiGeneBlast can be employed to identify BGCs within any genomic database like MIBiG, which comprises of known gene clusters (Medema and Fischbach, 2015). Specific tools like NaPDoS (Natural Product Domain Seeker) are used to identify distinct antibiotic gene clusters within the genome.

## Actinobacterial Genomics and BGCs

The recent advancements in next-generation sequencing (NGS) and genome mining have revealed that actinobacteria have a potential of synthesizing enormous compounds. The *Streptomyces* sp. within the group actinobacteria harbor linear chromosomes and have the largest genome among bacteria ranging from 6 to 12 Mb (Tidjani et al., 2019; Nicault et al., 2020). They are responsible for the production of two-third of the total antibiotics that are in clinical use today (Takahashi and Nakashima, 2018; Van Bergeijk et al., 2020). The production of these bioactive compounds depends on the presence of genes that encode the enzymes essential for synthesizing these compounds. These genes are arranged in groups known as BGCs in microbial genomes (Cimermancic et al., 2014).

Different studies have revealed that actinobacterial genomes are a storehouse of the plethora of antibiotics (Salwan and Sharma, 2020; Silva et al., 2020). *Streptomyces* are not the only source for the production of bioactive compounds, but they are prolific producers and therefore the genus is the greatest in the prokaryotes and also contains wide numbers of 20–30 BGCs for secondary metabolites, which include PKSs and NRPs. Total, 1346 BGCs detected by antiSMASH in the 39 streptomycete genomes in the antiSMASH database (https://antismash-db.secondarymetabolites.org/; accessed March 2018) (Ward and Allenby, 2018). Earlier, the discovery of BGC was based entirely on bioactive screening, which involves the laborious procedure of construction and screening the genome libraries. The advent of whole-genome sequencing methods revolutionized the detection of BGCs. *Streptomyces coelicolor*, before its genome sequencing, was known to produce four metabolites (Gomez-Escribano and Bibb, 2011; Hoskisson and Van Wezel, 2019). The whole-genome sequencing revealed the presence of 18 BGC, which were utterly incognito (Berdy, 2012). Since then, novel methods have been developed to detect BGCs using whole-genome databases and were termed genome mining (Albarano et al., 2020; Nicault et al., 2020; Van Bergeijk et al., 2020). The approach of genome mining enables prediction of BGCs within actinobacterial genome data by using bioinformatics tools like antiSMASH. Once the gene clusters are predicted they are made to express under laboratory conditions for the production of different novel compounds. Certain novel compounds were discovered recently using the genome mining approach, including humidimycin, isolated from *Streptomyces humidus* (Sánchez-Hidalgo et al., 2020), pentaminomycin from *Streptomyces cacaoi* (Kaweewan et al., 2020), pentamycin from *Streptomycin* sp. S816 (Zhou et al., 2019), salinosporamide A from marine actinobacteria *Salinospora tropica* (Pérez and Fenical, 2017), and abyssomicin C from a marine *Verrucosispora* (Freundlich et al., 2010; Table 1). Implementing a genome mining-based approach in *Streptomyces curacoi* resulted in the discovery of oxazole, methyloxazole, and thiazole ring containing macrocyclic compound named Curacozole (Kaweewan et al., 2020).

**TABLE 1:** Secondary metabolites production from actinobacteria with their bioactivities.

**Sl. No**	**Organism**	**Source**	**Compound name**	**Bioactivity**	**References**
1	*Nocardiopsis alba*	Soil	(Z)-1-((1-hydroxypenta-2,4-dien-1 yl)oxy)anthracene-9,10-dione	Antioxidant	Janardhan et al., 2014
2	*Nonomuraea specus*	Cave	Hypogeamicins	Cytotoxic	Derewacz et al., 2014
3	*Streptomyces* sp.	Soil	Huanglongmycin (HLM) A	Cytotoxic	Jiang et al., 2018
4	*Streptomyces* sp.	Cave	Chaxalacin B	Antimicrobial	Axenov-Gibanov et al., 2016
5	*Streptomyces* sp.	Soil	Pyridine-2,5-diacetamide	Antimicrobial and cytotoxic activity	Nithya et al., 2017
6	*Micromonospora neihuensis*	Soil	Neihumicin	Cytotoxic and antifungal activity	Wu et al., 1988
7	*Streptomyces* sp.	Soil	Isocoumarins, streptorubin B	Antimicrobial	Wu et al., 2016
8	*Salinispora tropica*	Marine	Salinosporamide A	Antimicrobial	Asolkar et al., 2010
9	*Saccharomonospora* sp.	Marine	Iodopyridone	Antimicrobial	Dias et al., 2012
10	*Streptomyces roseosporus*	–	Daptomycin	Antibacterial activity	Fenton et al., 2004
11	*Streptomyces hygroscopicus var ascomyceticus*	–	Pimecrolimus	Anticancer	Sugiura et al., 2005
12	*Streptomyces atratus*	Deep sea	Atratumycin	Antimicrobial	Sun et al., 2019
13	*Streptomyces albus*		Nybomycin	Antimicrobial	Rodríguez Estévez et al., 2020
14	*Streptomycin humidicus*	Soil	Humidimycin	Antimicrobial and anti-HIV activity	Sánchez-Hidalgo et al., 2020
15	*Streptomyces lydicus*		Netamycin	Antifungal	Wu et al., 2017

## Challenges in Genome-Guided NP Discovery

Even after genome mining and discovering unexpressed BGCs in actinobacteria, utilizing it for NP discovery remains a tedious challenge. *Streptomyces coelicolor* being the most studied actinobacteria, still harbors undiscovered biosynthetic pathways. These actinobacteria carried out the discovery of rare amicetin by inactivating the biosynthetic genes of streptomycin and streptothricin (Zhou et al., 2019). Programming a genome for the production of NPs is the biggest challenge in discovering novel bioactive compounds. Another challenge that needs to be considered is that even after choosing a BGC for desired NP production, its expression within the host or laboratory conditions remains poor (Lee et al., 2019). The actinobacteria produce metabolites by multi-enzyme catalytic pathways, which are encoded by BGCs. The biotic and abiotic stresses strictly regulate these gene clusters’ expression in their thriving natural habitat. Therefore, only a speck of metabolites is produced within the controlled environment of laboratories. To harness the full potential of actinobacteria for synthesis of diverse compounds; we need to understand the natural stimulus required to induce the production of NPs. The complete biosynthetic potential of actinobacteria in such a scenario can be explored by developing tools to detect complete BGCs, especially those that remain silent in laboratory conditions.

One of the critical steps in genome mining studies is the prediction of NP structures from the genome. Bioinformatics tools like antiSMASH (Blin et al., 2019) and PRISM (Skinnider et al., 2017) are available that ensures the structural novelty of the NPs. Still, another challenge is the precise prediction of these structures, as these tools are based on our current understanding of BGC knowledge. The prediction of these structures is based on the algorithm and the information within the available databases. The lack of genomic data affects the predictive accuracy of the NP structure. There exist 16 different tailoring pathways that combine to form the complex structure of erythromycin. The lack or improper prediction of any tailoring step may result in its inaccurate system (Amarasinghe et al., 2020).

## Harnessing Genomic Potential of Actinobacteria for NP Discovery

Most of the BGCs are silent or “cryptic,” which require advanced approaches like genome editing, genome engineering, or metabolic engineering for expression. The available datasets are point out that there is a plethora of uncharacterized BGC in nature whose potential is yet to be characterized (Lee et al., 2020). The hybrid clusters of PKSs, NRPs have been the current research array as an existing lot of available antibiotics have majorly become inefficacious against potential pathogens. The products formed by the combination of clusters can be crucial and might counter the problem of infections caused by resistant microbes by providing novel bioactive molecules. The production of virginiamycin and oxazolomycin has been reported recently, resulting from PKS/NRPS hybrid (Sharma and Thakur, 2020).

The bottlenecks in the biosynthetic pathways restrict the natural production of metabolites in high titers. There are different targeted approaches that are designed for the activation of specific BGCs in actinobacteria which includes heterologous expression, promoter exchange and BGC regulator manipulation. Heterologous expression of BGCs has become a common approach for NP discovery (Ren et al., 2017). This strategy requires a suitable host that can be genetically mutated and is fast growing. Among *Streptomyces* species, *S. coelicolor* (Zhou et al., 2012), *S. avermitilis* (Komatsu et al., 2010), *S. albus* have been genetically engineered for the expression of desired metabolites (Liu et al., 2018). *S. coelicolor* A(3)2 is the most commonly used heterologous host for expression and production of different classes of NPs like novobiocin (Eustáquio et al., 2005), salinomycin (Yin et al., 2015).

### Activation of Silent BGCs

Within the natural environment, the actinobacterial BGCs get expressed at a much better scale than that in laboratory conditions. The expression of silent or poorly expressed gene clusters can be regulated using transcriptional activators. The transcription mechanism of BGCs involves specific gene clusters comprising of activators and repressors, which can activate or suppress any biosynthetic pathway. Apart from activating the cryptic genes, transcriptional regulators can also upscale the production of actinobacterial metabolites (Doroghazi et al., 2014). The presence PSRs genes offer possibility of activating BGCs by suppressing the repressor or upscale production of metabolite or by over-expression of activator (Baral et al., 2018). The discovery of antibiotic argimycin P using *Streptomyces argillaceus* was possible only after inactivation of TetR transcriptional repressor gene arpRII (Ye et al., 2018). Similarly, the transcription regulation for overexpression of orf22 and orf42 in *Streptomyces fungicidicus* upscaled the production of enduracidins by 4-fold (Chen et al., 2019). Different approaches for activating the silent BGCs include optimization of organism’s growth media, which is a hit and trial approach—altering various sources of nutrition influences the organism’s metabolome, thus activating their BGCs. This approach has enabled the discovery of novel thiopeptide antibiotic TP-1161 from *Nocardiopsis* sp. Since actinobacteria dwell in soil, it is reported that addition of rare earth elements like scandium and lanthanum can enhance the production of antibiotic to multifold (Xia et al., 2020). Other techniques include the addition of elicitors, metabolic engineering, and ribosome engineering. Practically, γ-butyrolactones can be used as chemical elicitors for the production of antibiotics like virginiamycin, showdomycins in actinobacteria. Using ribosomal engineering, *S. coelicolor* A3(2) yielded 1.63 g/L of actinorhodin which is 180-fold higher than wild type strain (Sharma and Thakur, 2020).

The activation method of cryptic BGCs includes expression in heterologous hosts, promoter exchange, and genome editing using CRISPR (Li et al., 2018). The most commonly employed strategy is the isolation of BGC from the native environment and its heterologous expression within the host. This BGC activation strategy is most successful as the host can be domesticated in the characterized environment and is genetically stable.

### Expression in Heterologous Hosts

The novel BCGs identified and chosen to discover NP pose hurdles in expression within the standard fermentation process. The majority of the BCG are silent or cryptic, and heterologous expression is the only possible solution for the expression of these gene clusters. This expression is carried out in three significant steps: identifying BGC in the actinobacterial genome, cloning and expression in a heterologous host, and product isolation. There lies many constrains in heterologous expression of selective metabolite production. The major limitation is that only a few organisms can be used a model for heterologous expression (Demain, 2014). Apart from that mandatory knowledge of bias codons, substrate specificity and metabolic regulation of host should be available (Luo et al., 2015). The probability of expression of native promoters is always a question. Moreover, the expressed product may prove to be toxic for the heterologous host (Helfrich et al., 2014). Maintaining a fine tuning between the expression and production of metabolite should be in check. Evading these shortcomings lead to low recombination efficiency.

Within the natural environment, the actinobacterial genes are expressed better than its laboratory cultivation. Specific genes become cryptic or silent once actinobacteria are cultured on an artificial medium. To express these silent genes, a better understanding of actinobacterial interaction with the biotic and abiotic components of its environment is requisite (Lee et al., 2020).

### CRISPR Technology

Over a brief period, CRISPR/Cas9 genome editing tool has been a great breakthrough in manipulating different organisms’ genetic makeup (Tong et al., 2015). *Streptococcus thermophilus* which is widely used for making cheese and yogurts was often infected with virus which effected the quality of these food products. The alteration in genetic makeup using CRISPR sequences made *S. thermophilus* immune toward viral attacks. Huang et al. (2015) utilized CRISPR/Cas9 for removing BGCs of various lengths from the most widely investigated actinobacteria *S. coelicolor*. This tool is useful in engineering a host for heterologous expression and the production of NPs. Through the RNA guided DNA editing technology of CRISPR/Cas9 the BGCs of different length from *S. coelicolor* was removed and its heterologous expression was carried out in native host for the production of various novel compounds. Using CRISPR/Cas9 genes and BGCs of *Streptomyces pyogenes* were deleted, and site-directed mutagenesis was performed, which gradually enhanced the secondary metabolite production (Zeng et al., 2015). This technology was also utilized to activate and express silent BGCs in *Streptomyces* sp. (Zhang et al., 2017). This tool is highly efficient, and its combination with other existing technology can result in discovering novel compounds.

CRISPR-Cas9 mediated promoter knockout strategy is widely employed for activation of silent BGCs within actinobacteria. The novel amexanthomycins A-J were discovered only after knocking out rifampicin promoter genes in *Amycolatopsis mediterranei* S6991 (Hug et al., 2018). The native promoter within *S. albus* was replaced by a strong constitutive promoter using CRISPR-Cas9 (Li et al., 2018). This modification in the genome gradually enhanced metabolites’ biosynthesis, and novel compounds like type II polyketides were obtained using this technique (Zhang et al., 2017). The successful production of auroramycin, a type I PKS was carried out using *S. roseosporus* NRRL 15998 by CRISPR-Cas9 based promoter knock in of KasOp promoter to activate the silent BGCs (Lim et al., 2018). A similar approach was carried out in *S. venezuelae*, *S. lividans*, and *S. viridochromogenes*to discover NPs (Jia et al., 2017). The promoter knockout technique is useful in the activation and expression of silent BGCs. CRISPR-Cas9 is highly efficient in actinobacterial genome editing and has revolutionized the genome engineering technology to discover new uncharacterized compounds.

## Conclusion and Future Prospective

The genetic potential for the discovery of bioactive molecules by actinobacteria can be determined *via* genome mining. Sequencing actinomycete genomes provide useful information for inventing novel antimicrobial agents. The sequencing of rare actinobacteria genomes guided with bioinformatics analysis will open the door for scientists to explore the biochemical pathways and, consequently, discover novel bioactive molecules. This approach would contribute to more discoveries of natural antibiotics and therefore promote the pharmaceutical industry.

The discovery of NPs by harnessing the genomic potential of actinobacteria is making significant progress with time. Still, the rate of discovery of novel bioactive compounds is lesser than the pace by which microbes are attaining resistance. To enhance the pace screening of BGCs and determining their novelty at the earliest is necessary. Moreover, a multidisciplinary approach involving microbiologists, computational biologists, and chemists needs to collaborate to increase the discovery of NPs from actinobacterial sources. The new environmental niches should be approached for isolation of novel actinobacteria, which might provide novel BCGs to discover NPs.

Various researchers reported that actinobacteria especially genus *Streptomyces* and rare actinobacteria are well-known to produce wide number of bioactive compounds (Barka et al., 2016; Genilloud, 2017; Li et al., 2019a). In the last 10 years, a number of novel metabolic engineering and synthetic biology strategies have been established to exploit the development potential of actinobacteria, including dynamic metabolic control, BGC amplification, pathway refactoring, and genome-minimized *Streptomyces* chassis (Beites and Mendes, 2015; Tan and Liu, 2017; Li et al., 2019b). We anticipate that NP discovery and development will be accelerated rapidly by refactoring and amplifying entire biosynthetic pathways in combination with powerful heterologous expression platforms. It is very much essential to search diverse heterologous hosts and universal refactoring methods to trigger silent BGCs or for the production of secondary metabolites.

## Author Contributions

TS, AP, AJ, AH, and AA: writing original draft. SB, VG, and EA: writing-review and editing. All authors contributed to the article and approved the submitted version.

## Conflict of Interest

The authors declare that the research was conducted in the absence of any commercial or financial relationships that could be construed as a potential conflict of interest.
